# Pregnancy and Vaginal Delivery after Sacrohysteropexy

**DOI:** 10.1155/2015/305107

**Published:** 2015-06-23

**Authors:** Deniz Balsak, Ahmet Eser, Onur Erol, Derya Deniz Altıntaş, Şerif Aksin

**Affiliations:** ^1^Department of Obstetric and Gynecology, Haliç University, Faculty of Medicine, 21400 Istanbul, Turkey; ^2^Department of Obstetrics and Gynaecology, Zeynep Kamil Research and Training Hospital, Istanbul, Turkey; ^3^Department of Obstetrics and Gynaecology, Antalya Research and Training Hospital, Antalya, Turkey; ^4^Department of Radiology, Diyarbakır Research and Training Hospital, Diyarbakir, Turkey; ^5^Department of Obstetrics and Gynaecology, Diyarbakır Maternity and Children Hospital, Diyarbakır, Turkey

## Abstract

Pregnancy and birth after a Pelvic Organ Prolapse (POP) surgery is a rare condition and less is known about the method for delivery. A 31-year-old women with gravida 3 para 3 underwent abdominal sacrohysteropexy and transobturatuar tape (TOT) procedures for stage III prolapse who delivered via vaginal birth and showed no relapse. Sacrohysteropexy is a good option for women with POP who desire fertility with a long term follow-up period.

## 1. Introduction

Pelvic Organ Prolapse (POP) is the herniation of the pelvic organs which is one of the most causes of the benign gynecologic operations [1]. Uterus, cervix, bladder, small bowel, rectum, and vaginal walls can be affected in POP. There are different types of POP according to the location of pelvic floor defect and one of which is apical prolapse consisting of uterus and cervix.

Although conservative treatment provides benefits in POP, severe POP is treated by surgical methods including vaginal, abdominal, laparoscopic, or robotic interventions [2]. Patient's age, expectations, and fertility desires are prompted surgeons for uterine sparing prolapse surgeries [3].

This presentation is the first case report of a woman that had a subsequent pregnancy and vaginal birth after sacrohysteropexy.

Informed consent has been obtained for this report.

## 2. Case

A 31 year-old women gravida 3 para 3 was referred to our hospital with stage III POP and stress urinary incontinence (SUI). Her obstetric history was unremarkable and consisted of three vaginal deliveries in March 2012. The Pelvic Organ Prolapse Quantification (POP-Q) measurements were Aa: +3 Ba: +3 Ap: +3 Bp: +3 C: +2, D: +3, and TVL: 7 cm PB: 3 cm GH: 4 cm. She had been trying vaginal pessary and pelvic floor strengthening exercises, but she decided to have surgical intervention due to sexual discomfort, fertility desire, and no benefits with conservative therapy.

She underwent abdominal sacrohysteropexy and transobturator tape (TOT) procedures in May 2009. Procedure steps were as follows. First, presacral area was dissected to expose anterior longitudinal ligament. Next, rectovaginal and vesicovaginal spaces were dissected and then a Y shaped light polypropylene mesh was inserted in these dissected segments. Further, branched segment of mesh was sutured into the cervix anteriorly and into the rectovaginal space posteriorly by using 2-0 nonabsorbable PROLENE sutures (2-0 polypropylene suture, Ethicon). The other part of mesh was attached to the anterior longitudinal ligament. There was unremarkable bleeding. Sacrouterine plication and TOT procedure were then performed. In postoperative 3rd month, POP-Q measurements were Aa: −2, Ba: −1, Ap: −3, Bp −2, C: −4, D: −5, TVL: 8 cm, PB: 3 cm, and GH: 4 cm.

**Figure 1:**
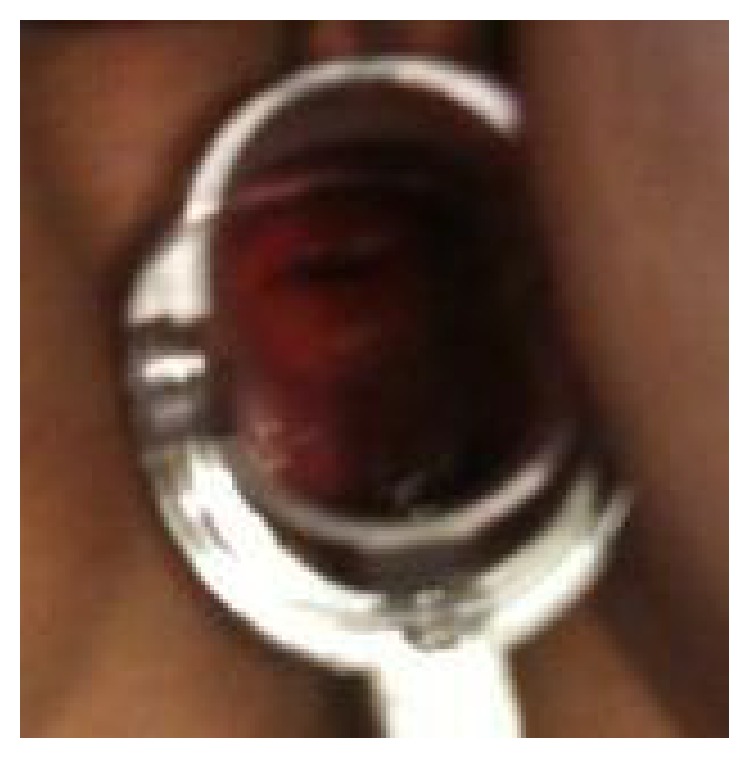
Cervical position at 12 months postpartum.

**Figure 2:**
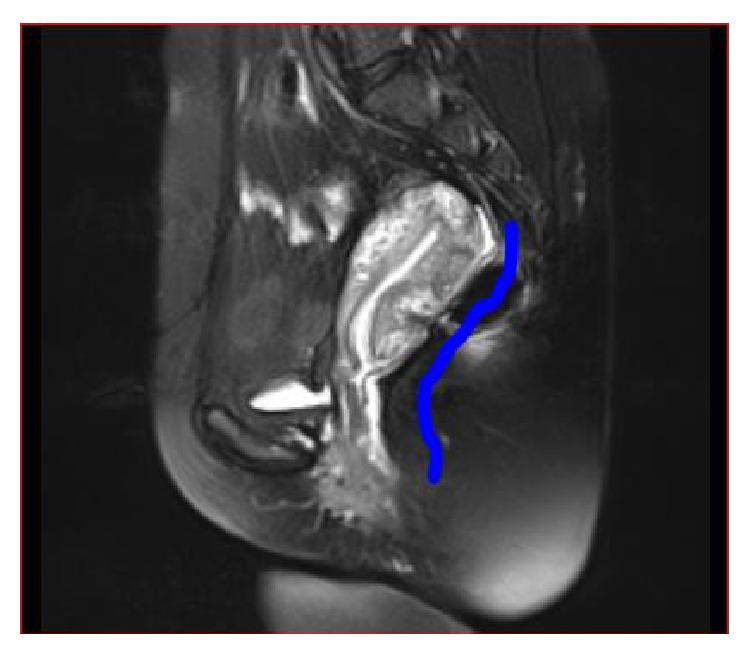
MRI appearance of the pelvis with uterus at 12 months postpartum.

Consequently, she underwent delivery 35 months after the procedure. She was referred to our delivery unıt with fully dilated cervix and the second stage of labor was 15 minutes. She gave birth to a 3950 gr. healthy newborn. 12 months after delivery, POP-Q measurements were Aa: −1, Ba: −2, Ap: −3, Bp: −2, C: −4, D: −5, TVL: 8 cm, PB: 3 cm, and GH: 4 cm, showing no POP and no stress incontinence recurrence (Figures 1 and 2).

## 3. Discussion

Surgical procedures for POP include vaginal, abdominal, and minimal invasive techniques such as laparoscopic and robotic sacropexy operations that have been performed [2]. Sacrocolpopexy is considered the gold standard treatment to correct POP [4]. Moreover, abdominal sacrocolpopexy is the most common used technique to repair prolapse.

POP can be seen due to fertile age, and uterine sparing surgery is becoming more important for women fertility desire and sexual function [5]. Although surgeons have performed uterine sparing surgery for POP, there is no study of how to manage delivery following a POP repair procedure. There are only two cases reported by Lewis and Culligan and Albowitz et al. in which the patients had POP surgery and followed subsequent pregnancy and cesarean deliveries. Lewis and Culligan reported a case in which a woman underwent pregnancy after six months of POP surgery and she delivered via caesarian section at term [6]. She reprolapsed after two years postpartum. In the case reported by Albowitz et al., a woman had no POP recurrence after the POP surgery and following delivery via caesarian section after 3 months of delivery [7]. According to these reports, long term follow-up period is necessary after the delivery.

Our patient was the first case to deliver via vaginal birth after the POP surgery and had no relapse after 12 months postpartum. If women desire fertility with POP, sacrohysteropexy regardless of a surgical technique (abdominal or minimal invasive methods) is an effective and reasonable treatment.

## 4. Conclusion

Pregnancy after an abdominal sacrohysteropexy would be an option for women who desire fertility without an increased risk of POP and normal vaginal delivery would be tried after the POP surgery. Further studies are required to determine the delivery method after POP surgery.
